# Right Ventricular Wound And Complete Mammary Artery Transection

**DOI:** 10.5935/abc.20180185

**Published:** 2018-09

**Authors:** Gregorio Laguna, Miriam Blanco, Cristina García-Rico, Yolanda Carrascal

**Affiliations:** Instituto de Ciencias del Corazon (ICICOR), Hospital Clinico Universitario Valladolid, Valladolid - Spain

**Keywords:** Stab Wounds/heart, Suicide, Attempted, Heart Injuries/cirurgia

Many patients die immediately after suffering a heart wound; on the other hand, many others die before the surgery, during surgery or later, due to complications.^1^

**Figure 1:**
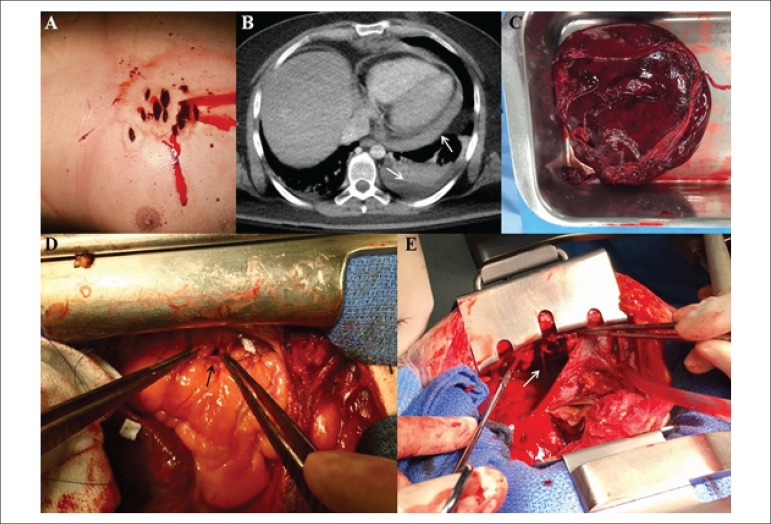
Panel A: Eleven knife-wounds localized in the left-anterior chest wall. Panel B: Axial computed tomography showed severe pericardial and left pleural effusion (white arrows). Panel C: The clot drained from the pericardial cavity. Panel D: Right ventricular perforation repaired using a monofilament suture (black arrow). Panel E: Complete mammary artery transection bleeding into left pleural cavity (white arrow).

We admitted a 33-year-old man after a suicide attempt occurring one hour before, with eleven knife-wounds localized in the left-anterior chest wall (Figure:1-A). Physical exam showed hypotension, dyspnea, high central venous pressure and mild external bleeding. Hemodynamic monitoring, tracheal intubation, vasopressor perfusion, fluid therapy and urgent echocardiogram and tomography were undertaken. ACT showed severe pericardial effusion and moderate left pleural effusion (Figure:1-B, white arrows). Emergency cardiac surgery was performed through median sternotomy. Multiple pericardial tears were visualized. The pericardial clot was removed (Figure:1-C) and the right ventricular wound was closed using a monofilament suture (Figure:1-D, black arrow). In the inner chest wall, a complete left mammary artery transection was observed with severe bleeding into the left pleural cavity (Figure:1-E, white arrow). The mammary artery was repaired, and the bleeding was controlled. The postoperative course was uneventful.

Heart wounds are serious health problems. The dramatic statistics have shown that many problems connected with traumatic cardiac lesions are not ultimately resolved. Knife stabs to the right ventricle are perhaps the most common penetrating injury to the heart, but the additional complete transection of the mammary artery is very uncommon. The most important factor for survival is the urgency treatment and the immediate surgical repair.
